# Enhancing Outcomes: Acceptability of Medication Formulations for the Treatment of Acute Agitation in a Psychiatric Population

**DOI:** 10.3390/pharmacy11010004

**Published:** 2022-12-23

**Authors:** Rachel E. Walker, Leigh Anne Nelson, Carrie Kriz, Courtney A. Iuppa, Yifei Liu, Lauren A. Diefenderfer, Ellie S. R. Elliott, Roger W. Sommi

**Affiliations:** 1 Kansas City VA Medical Center, 4801 Linwood Boulevard, Kansas City, MO 64128, USA; 2 University of Missouri-Kansas City School of Pharmacy, Division of Pharmacy Practice and Administration, 2464 Charlotte Street, Kansas City, MO 64108, USA; 3 Center for Behavioral Medicine, 1000 E. 24th Street, Kansas City, MO 64108, USA; 4 Center for Behavioral Medicine & Northwest Missouri Psychiatric Rehabilitation Center, 1000 E. 24th Street, Kansas City, MO 64108, USA

**Keywords:** agitation, acceptability, schizophrenia, bipolar disorder, formulation

## Abstract

**BACKGROUND:** There is limited research evaluating patient acceptability of medication formulations in the treatment of acute agitation. This study assessed patient acceptability of medication formulations (tablet, orally-dissolving-tablet [ODT], liquid, intramuscular injection [IM], inhaled device [INH]) for the treatment of acute agitation and examined correlating factors. **METHODS:** Adults with psychotic illness or bipolar disorder receiving emergency or inpatient services at an inpatient psychiatric facility in Kansas City, Missouri were included. Participants viewed a presentation on medication formulations for acute agitation and were surveyed on acceptability (measured on a five-point Likert scale). The primary outcome variable was the attitudinal measurement of acceptability of each formulation in correlation with the severity of agitation for use in themselves and other patients. **RESULTS:** One hundred participants completed the survey. Participants rated the following: (1) This medication formulation would be acceptable to treat mild agitation in themselves and others (oral tablet 85% and 48%; ODT 82% and 55%; liquid 74% and 51%; IM 53% and 74%; INH 78% and 72%); and (2) This medication formulation would be acceptable to treat severe agitation in themselves and others (oral tablet 75% and 52%; ODT 74% and 53%; liquid 66% and 53%; IM 61% and 67%; INH 77% and 72%). For treating mild agitation, participants preferred tablets and ODTs to the IM (*p* < 0.05) and the INH to liquid or IM (*p* < 0.05), for themselves; and oral formulations were preferred to the IM (*p* < 0.05) for other patients. For severe agitation in themselves and others, preference for the INH and IM versus oral formulations (*p* < 0.05) was significant, with no difference between the INH and IM (*p* > 0.05). **CONCLUSIONS:** The proportion of responses preferring oral formulations was higher than IM and INH. Dosage formulation acceptability differed depending on the severity of agitation and intended recipient of the medication.

## 1. Background

Although an emphasis on the reduction of symptom burden is a primary goal for the treatment of psychiatric illnesses, treatment guidelines now advocate for the inclusion of patient preference in drug selection (shared decision making or informed choice) [1,2,3]. A survey of inpatients with schizophrenia conducted in 2010 found that 42% wished to participate in treatment decisions [4]. Patients who participated in treatment decisions had an improved therapeutic alliance, more positive attitudes regarding drug therapy, and improved insight compared to patients who had not participated.^4^ In addition, lack of patient participation in decisions regarding antipsychotic treatment has been correlated with decreased patient satisfaction and may lead to nonadherence [5,6].

Agitation and aggression are behavioral manifestations that can be serious, and range in complexity and difficulty of treatment [7]. It is challenging to include patient input when acutely treating behavioral emergencies. In emergency and inpatient psychiatry settings, agitation is commonly treated with antipsychotics. These medications may be administered orally (oral tablets/capsules, orally dissolving tablets, sublingual tablets or liquid), intramuscularly, intravenously or by inhalation. The Expert Consensus Guidelines on the Treatment of Behavioral Emergencies recommend that patient preference be included in the decision-making process for both the choice of medication and route of administration when treating agitation [8]. The incorporation of the patient’s choice of medication and drug formulation during an episode of agitation may have important implications for a reduced side-effect risk, an improved medication acceptability and adherence, and the establishment of a good therapeutic alliance.

There is little information regarding patient preference of medication or formulation in the treatment of acute agitation. An online survey conducted in Denmark and Sweden in 2014 evaluated patient preference of medication formulation for the treatment of agitation in an adult population with a self-reported diagnosis of schizophrenia or bipolar disorder [9,10]. After being educated on the different treatment routes, 66% of survey respondents preferred treatment with an inhaler, compared with 21% preferring tablets and 13% preferring an injection [10]. Treatment with an inhaler was also associated with an increase in respondent-assessed quality-adjusted life years (QALYs) compared to treatment with tablets or an injection [9].

Few studies exist on psychiatric patients’ preferences for treatment of acute agitation, and none have been conducted in the United States. The primary objective of this study was to elicit the patient-rated acceptability of different medication formulations in correlation with the severity of an acute agitation episode and whether the medication was administered to that participant as compared to another person.

## 2. Methods

This single-center, prospective, cross-sectional survey study measured the acceptability of five medication formulations used in the treatment of acute agitation. The study took place over eight weeks and included 100 participants voluntarily hospitalized at University Health (formerly Truman Medical Center) Behavioral Health emergency department and inpatient psychiatry units in Kansas City, Missouri. English-speaking adults with a diagnosis of psychotic illness (schizophrenia, schizoaffective disorder, psychosis not otherwise specified) or bipolar disorder and without a comorbid diagnosis of severe intellectual disability were invited to participate. Patients with a legal guardian or hospitalized by court order were excluded. All participants provided informed consent.

To standardize participants’ knowledge of agitation and medication formulations used in its treatment, all participants viewed a recorded presentation prior to survey completion. The presentation defined agitation, gave examples of agitation severity, and reviewed 5 medication formulations (oral tablet, orally dissolving tablet [ODT), liquid medication, intramuscular [IM) injection, and inhaler [INH) device) used to treat agitation and their associated administration method and onset of effect. Participants were then asked to complete a survey addressing their perceptions and preferences for each medication formulation used to treat an episode of acute agitation. The presentation and survey were both completed with the use of a computer tablet, and assistance in completing the questionnaire was offered.

The survey assessed participants’ preference of medication formulation acceptability. The survey was divided into six segments: one for each of the five medication formulations and patient sociodemographic information. For each of the formulations, participants rated five statements on the acceptability of the formulation for both themselves and other patients under conditions of mild and severe agitation. The degree with which the subject agreed with each statement was measured on a 5-point Likert scale ranging from “Strongly Agree” to “Strongly Disagree”. The subject was then asked to choose one formulation as their overall preference in the treatment of an episode of mild agitation and one for the treatment of a severe episode of agitation. The sociodemographic factors collected included: age, gender, ethnicity, marital status, highest level of education, current living situation, current employment status, primary psychiatric diagnosis, previous psychiatric hospitalizations in the past twelve months, and duration of mental illness.

Subjects that completed the survey received $10.00 in cash as compensation for their time. The study was approved by the University of Missouri—Kansas City (UMKC) Institutional Review Board. Data were collected and managed using REDCap (Research Electronic Data Capture) hosted at the UMKC Center for Health Insights [11].

Descriptive statistics and a paired sample t-test were used to assess for differences between medication formulations. The five-point Likert scale was weighted with 1 for “strongly disagree” up to 5 for “strongly agree” to stratify results appropriately.

## 3. Results

One hundred participants were enrolled in this study and included in the analysis. Sociodemographic characteristics are found in Table 1. The population consisted mostly of males (75%) between the ages of 18–40 years (63%) who were white (47%) and unemployed (81%). A majority of participants had a psychotic illness (62%), and half were diagnosed with a mental illness more than 10 years prior to the study participation (50%).

**Figure 1:**
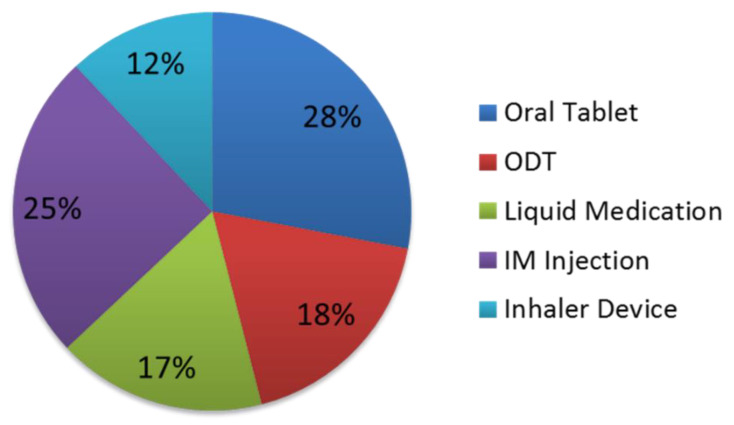
Proportions of responses indicating overall preference for medication formulation for the treatment of mild agitation.

**Figure 2:**
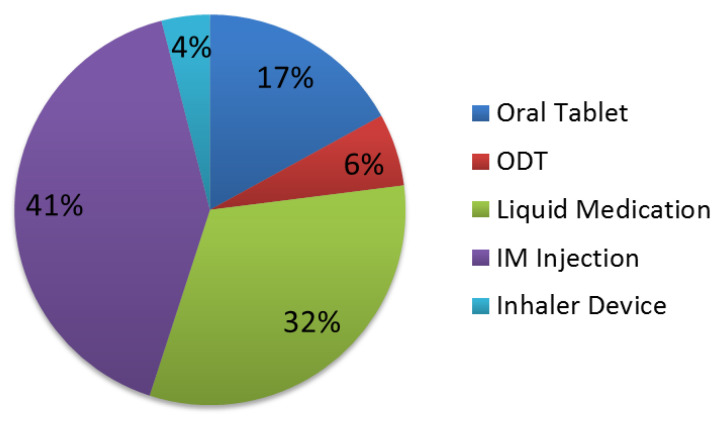
Proportions of responses indicating overall preference for medication formulation for the treatment of severe agitation.

The proportion of responses preferring oral formulations was higher than IM and INH (Figure 1 and Figure 2). Preference for an oral medication formulation decreased in severe agitation from 63% to 55%, while preference for an IM injection increased from 25% to 41%. Preference for different types of oral medication formulations also varied due to the severity of agitation.

Survey questions assessed the medication formulation preference for both mild and severe agitation in the participant and other patients. Drug formulation preference for patients other than themselves during an episode of mild agitation indicated that an IM was significantly less acceptable than all other medication formulations, and there was a preference for these individuals to receive an oral tablet over a liquid medication. Responses differed when asked about acceptable medication formulations for themselves during an episode of mild agitation, with participants preferring an oral tablet to IM; an IM over an ODT; and an INH as compared to a liquid medication or an IM. When asked about a severe episode of agitation (destroying property or hurting other people), participants preferred medications with a more rapid onset for another patient, favoring an IM over oral tablet, ODT, or liquid medication, and an INH over liquid medication or an oral tablet. Participants similarly favored medication formulations with a rapid onset when asked about acceptance of a medication for themselves in an episode of severe agitation, with a preference for an INH and IM over all other formulations (*p* < 0.05).

Additionally, all responses for the five statements for each formulation were collapsed to indicate the overall attitude. A significant correlation was seen between this variable of overall attitude and the choice for the use of an oral tablet during a severe episode of agitation. If participants had received a medication to treat agitation during the current hospitalization, they were asked about their role in its selection. Forty-five participants met this criterion and reported that the physician chose the medication in 69% (*n* = 31) of the cases, while 29% (*n* = 13) reported having an input in the decision and 2% (*n* = 1) reported sole autonomy in the choice.

## 4. Discussion

Behavioral emergencies are universally known to be challenging conditions to appropriately treat, and psychiatric patients often have limited participation in medication and formulation selection due to the acuity of the situation. There is inadequate research evaluating patient preference and acceptability of medication formulations for the treatment of acute agitation. A survey study conducted in Denmark and Sweden found that patients with schizophrenia or bipolar disorder reported injections to be the least preferred formulation for treatment of agitation [10]. Preference for an INH was highest at 44%, followed by oral tablets at 41% and then IM at 16%. When these participants were educated about the delivery method, including the time to onset of effect, risk and duration of adverse effects, an INH was preferred by 66% of participants, oral tablets by 21%, followed by an IM by 13%. Another survey study conducted by the same authors found an INH to be the preferred administration method for treatment of agitation, indicated by the highest QALY, and found IM to be the least valuable route of administration, indicated by the lowest QALY [9].

To our knowledge, our study is the first conducted in the United States to evaluate the acceptability of different medication formulations in the management of acute agitation. Similar to the Nordic studies, IM was the least acceptable medication formulation in our study for the management of agitation, regardless of agitation severity, but oral formulations were preferred over an INH. Participants in our study perceived the need for a faster onset of efficacy in severe agitation, as the overall acceptability for IM increased but the acceptability of an INH decreased from 12% to 4%. Acceptability of a medication formulation for both mild and severe agitation differed depending on whether they themselves were the recipients of the medication or the recipient was another patient. The importance of patients’ input into their treatment decisions has been addressed in treatment guidelines and is integral to improving health care and the appropriate management of episodes of agitation. Our study found that 45% of participants had received pharmacologic treatment for agitation during the current hospitalization and that only a minority reported having input into the treatment decision. This affirms the notion that psychiatric patients have little influence on the medication and/or formulation prescribed to treat their acute agitation in an inpatient setting.

A limitation of this study is the lack of generalizability; thus, the results may not be transferable to all patients in the United States or other countries. More severely ill or treatment-resistant patients with schizophrenia and bipolar disorder were not included in this study, as patients hospitalized involuntarily or voluntarily by a guardian were excluded from participation. Patients were not approached for study participation if they were acutely agitated or had received a medication to treat agitation within the last 8 h. These patients were approached regarding study participation at a later time during their hospitalization. A validated survey tool or questionnaire was not used to measure acceptability, although a statistical measure was applied to our question set and affirmed the validation and reliability of the instrument.

## 5. Conclusions

This is the first study conducted in the United States to report the acceptability of different medication formulations for the management of acute agitation episodes with schizophrenia and bipolar disorder. The proportion of responses preferring oral formulations was higher than IM and INH. Medication formulation acceptability differed depending on the severity of agitation and the intended recipient of the medication.

## Figures and Tables

**Table 1:** Demographic characteristics.

Characteristic	% (*n*)
**Total Sample**	100% (100)
**Male**	75% (75)
**Age**	
18–40 years	63% (63)
41–65 years	37% (37)
**Ethnicity**	
• White/Caucasian	47% (47)
• Black or African American	38% (38)
• Other	15% (15)
**Primary Psychiatric Diagnosis**	
• Bipolar Disorder	38% (38)
• Schizophrenia	30% (30)
• Psychosis NOS	18% (18)
• Schizoaffective Disorder	14% (14)
**Marital Status**	
• Single	70% (70)
• Other (Separated, Divorced or Widowed)	17% (17)
• Married/Domestic Partnership	13% (13)
**Current Level of Employment**	
• Not employed	81% (81)
• Full-Time	10% (10)
• Part-Time	9% (9)
**Highest Level of Education**	
• Attended grade school or completed 8th grade	5% (5)
• Attended high school, no diploma	21% (21)
• Completed high school, diploma or GED	35% (35)
• Attended some college, no degree	27% (27)
• Completed college, degree	12% (12)
**Current Living Situation**	
• Living alone—independent living situation	30% (30)
• Homeless shelter or living on the street	29% (29)
• Family of origin—parents, grandparents, siblings or other relatives	15% (15)
• Family of orientation—spouse, children	10% (10)
• Living with friends	6% (6)
• Structured environment—board and care, group home, halfway house	5% (5)
• Other living situation	5% (5)
**Number of Hospital or ED Visits Due to Psychiatric Illness in the Past 12 Months**	
• 1	25% (25)
• 2	20% (20)
• 3	17% (17)
• 4	14% (14)
• 5	5% (5)
• 6–9	14% (14)
• 10 or more	5% (5)
**Duration of Mental Illness**	
• Less than 1 year	6% (6)
• 1–2 years	13% (13)
• 3–5 years	12% (12)
6–10 years	19% (19)
• 11 years or more	50% (50)

## Data Availability

Not applicable.
